# Supplementary material for: Bayesian refinement of protein structures and ensembles against SAXS data using molecular dynamics
Source: PLoS Comput Biol. 2017 Oct 18;13(10):e1005800. doi: 10.1371/journal.pcbi.1005800 (PMC5662244; doi:10.1371/journal.pcbi.1005800)
Supplement: S1 Archive — (BZ2) [file pcbi.1005800.s011.tar.bz2 › gromacs_4.62_bayesian_saxs_refi/share/html/online/mdrun.html]

mdrun


| mdrun Main Table of Contents |

**VERSION 4.6  
Sat 19 Jan 2013**

---

### Description

The mdrun program is the main computational chemistry engine
within GROMACS. Obviously, it performs Molecular Dynamics simulations,
but it can also perform Stochastic Dynamics, Energy Minimization,
test particle insertion or (re)calculation of energies.
Normal mode analysis is another option. In this case mdrun
builds a Hessian matrix from single conformation.
For usual Normal Modes-like calculations, make sure that
the structure provided is properly energy-minimized.
The generated matrix can be diagonalized by g\_nmeig.

The mdrun program reads the run input file (-s)
and distributes the topology over nodes if needed.
mdrun produces at least four output files.
A single log file (-g) is written, unless the option
-seppot is used, in which case each node writes a log file.
The trajectory file (-o), contains coordinates, velocities and
optionally forces.
The structure file (-c) contains the coordinates and
velocities of the last step.
The energy file (-e) contains energies, the temperature,
pressure, etc, a lot of these things are also printed in the log file.
Optionally coordinates can be written to a compressed trajectory file
(-x).

The option -dhdl is only used when free energy calculation is
turned on.

A simulation can be run in parallel using two different parallelization
schemes: MPI parallelization and/or OpenMP thread parallelization.
The MPI parallelization uses multiple processes when mdrun is
compiled with a normal MPI library or threads when mdrun is
compiled with the GROMACS built-in thread-MPI library. OpenMP threads
are supported when mdrun is compiled with OpenMP. Full OpenMP support
is only available with the Verlet cut-off scheme, with the (older)
group scheme only PME-only processes can use OpenMP parallelization.
In all cases mdrun will by default try to use all the available
hardware resources. With a normal MPI library only the options
-ntomp (with the Verlet cut-off scheme) and -ntomp\_pme,
for PME-only processes, can be used to control the number of threads.
With thread-MPI there are additional options -nt, which sets
the total number of threads, and -ntmpi, which sets the number
of thread-MPI threads.
Note that using combined MPI+OpenMP parallelization is almost always
slower than single parallelization, except at the scaling limit, where
especially OpenMP parallelization of PME reduces the communication cost.
OpenMP-only parallelization is much faster than MPI-only parallelization
on a single CPU(-die). Since we currently don't have proper hardware
topology detection, mdrun compiled with thread-MPI will only
automatically use OpenMP-only parallelization when you use up to 4
threads, up to 12 threads with Intel Nehalem/Westmere, or up to 16
threads with Intel Sandy Bridge or newer CPUs. Otherwise MPI-only
parallelization is used (except with GPUs, see below).

To quickly test the performance of the new Verlet cut-off scheme
with old .tpr files, either on CPUs or CPUs+GPUs, you can use
the -testverlet option. This should not be used for production,
since it can slightly modify potentials and it will remove charge groups
making analysis difficult, as the .tpr file will still contain
charge groups. For production simulations it is highly recommended
to specify cutoff-scheme = Verlet in the .mdp file.

With GPUs (only supported with the Verlet cut-off scheme), the number
of GPUs should match the number of MPI processes or MPI threads,
excluding PME-only processes/threads. With thread-MPI the number
of MPI threads will automatically be set to the number of GPUs detected.
When you want to use a subset of the available GPUs, you can use
the -gpu\_id option, where GPU id's are passed as a string,
e.g. 02 for using GPUs 0 and 2. When you want different GPU id's
on different nodes of a compute cluster, use the GMX\_GPU\_ID environment
variable instead. The format for GMX\_GPU\_ID is identical to
-gpu\_id, but an environment variable can have different values
on different nodes of a cluster.

When using PME with separate PME nodes or with a GPU, the two major
compute tasks, the non-bonded force calculation and the PME calculation
run on different compute resources. If this load is not balanced,
some of the resources will be idle part of time. With the Verlet
cut-off scheme this load is automatically balanced when the PME load
is too high (but not when it is too low). This is done by scaling
the Coulomb cut-off and PME grid spacing by the same amount. In the first
few hundred steps different settings are tried and the fastest is chosen
for the rest of the simulation. This does not affect the accuracy of
the results, but it does affect the decomposition of the Coulomb energy
into particle and mesh contributions. The auto-tuning can be turned off
with the option -notunepme.

mdrun pins (sets affinity of) threads to specific cores,
when all (logical) cores on a compute node are used by mdrun,
even when no multi-threading is used,
as this usually results in significantly better performance.
If the queuing systems or the OpenMP library pinned threads, we honor
this and don't pin again, even though the layout may be sub-optimal.
If you want to have mdrun override an already set thread affinity
or pin threads when using less cores, use -pin on.
With SMT (simultaneous multithreading), e.g. Intel Hyper-Threading,
there are multiple logical cores per physical core.
The option -pinstride sets the stride in logical cores for
pinning consecutive threads. Without SMT, 1 is usually the best choice.
With Intel Hyper-Threading 2 is best when using half or less of the
logical cores, 1 otherwise. The default value of 0 do exactly that:
it minimizes the threads per logical core, to optimize performance.
If you want to run multiple mdrun jobs on the same physical node,you should set -pinstride to 1 when using all logical cores.
When running multiple mdrun (or other) simulations on the same physical
node, some simulations need to start pinning from a non-zero core
to avoid overloading cores; with -pinoffset you can specify
the offset in logical cores for pinning.

When mdrun is started using MPI with more than 1 process
or with thread-MPI with more than 1 thread, MPI parallelization is used.
By default domain decomposition is used, unless the -pd
option is set, which selects particle decomposition.

With domain decomposition, the spatial decomposition can be set
with option -dd. By default mdrun selects a good decomposition.
The user only needs to change this when the system is very inhomogeneous.
Dynamic load balancing is set with the option -dlb,
which can give a significant performance improvement,
especially for inhomogeneous systems. The only disadvantage of
dynamic load balancing is that runs are no longer binary reproducible,
but in most cases this is not important.
By default the dynamic load balancing is automatically turned on
when the measured performance loss due to load imbalance is 5% or more.
At low parallelization these are the only important options
for domain decomposition.
At high parallelization the options in the next two sections
could be important for increasing the performace.

When PME is used with domain decomposition, separate nodes can
be assigned to do only the PME mesh calculation;
this is computationally more efficient starting at about 12 nodes.
The number of PME nodes is set with option -npme,
this can not be more than half of the nodes.
By default mdrun makes a guess for the number of PME
nodes when the number of nodes is larger than 11 or performance wise
not compatible with the PME grid x dimension.
But the user should optimize npme. Performance statistics on this issue
are written at the end of the log file.
For good load balancing at high parallelization, the PME grid x and y
dimensions should be divisible by the number of PME nodes
(the simulation will run correctly also when this is not the case).

This section lists all options that affect the domain decomposition.

Option -rdd can be used to set the required maximum distance
for inter charge-group bonded interactions.
Communication for two-body bonded interactions below the non-bonded
cut-off distance always comes for free with the non-bonded communication.
Atoms beyond the non-bonded cut-off are only communicated when they have
missing bonded interactions; this means that the extra cost is minor
and nearly indepedent of the value of -rdd.
With dynamic load balancing option -rdd also sets
the lower limit for the domain decomposition cell sizes.
By default -rdd is determined by mdrun based on
the initial coordinates. The chosen value will be a balance
between interaction range and communication cost.

When inter charge-group bonded interactions are beyond
the bonded cut-off distance, mdrun terminates with an error message.
For pair interactions and tabulated bonds
that do not generate exclusions, this check can be turned off
with the option -noddcheck.

When constraints are present, option -rcon influences
the cell size limit as well.
Atoms connected by NC constraints, where NC is the LINCS order plus 1,
should not be beyond the smallest cell size. A error message is
generated when this happens and the user should change the decomposition
or decrease the LINCS order and increase the number of LINCS iterations.
By default mdrun estimates the minimum cell size required for P-LINCS
in a conservative fashion. For high parallelization it can be useful
to set the distance required for P-LINCS with the option -rcon.

The -dds option sets the minimum allowed x, y and/or z scaling
of the cells with dynamic load balancing. mdrun will ensure that
the cells can scale down by at least this factor. This option is used
for the automated spatial decomposition (when not using -dd)
as well as for determining the number of grid pulses, which in turn
sets the minimum allowed cell size. Under certain circumstances
the value of -dds might need to be adjusted to account for
high or low spatial inhomogeneity of the system.

The option -gcom can be used to only do global communication
every n steps.
This can improve performance for highly parallel simulations
where this global communication step becomes the bottleneck.
For a global thermostat and/or barostat the temperature
and/or pressure will also only be updated every -gcom steps.
By default it is set to the minimum of nstcalcenergy and nstlist.

With -rerun an input trajectory can be given for which
forces and energies will be (re)calculated. Neighbor searching will be
performed for every frame, unless nstlist is zero
(see the .mdp file).

ED (essential dynamics) sampling and/or additional flooding potentials
are switched on by using the -ei flag followed by an .edi
file. The .edi file can be produced with the make\_edi tool
or by using options in the essdyn menu of the WHAT IF program.
mdrun produces a .xvg output file that
contains projections of positions, velocities and forces onto selected
eigenvectors.

When user-defined potential functions have been selected in the
.mdp file the -table option is used to pass mdrun
a formatted table with potential functions. The file is read from
either the current directory or from the GMXLIB directory.
A number of pre-formatted tables are presented in the GMXLIB dir,
for 6-8, 6-9, 6-10, 6-11, 6-12 Lennard-Jones potentials with
normal Coulomb.
When pair interactions are present, a separate table for pair interaction
functions is read using the -tablep option.

When tabulated bonded functions are present in the topology,
interaction functions are read using the -tableb option.
For each different tabulated interaction type the table file name is
modified in a different way: before the file extension an underscore is
appended, then a 'b' for bonds, an 'a' for angles or a 'd' for dihedrals
and finally the table number of the interaction type.

The options -px and -pf are used for writing pull COM
coordinates and forces when pulling is selected
in the .mdp file.

With -multi or -multidir, multiple systems can be
simulated in parallel.
As many input files/directories are required as the number of systems.
The -multidir option takes a list of directories (one for each
system) and runs in each of them, using the input/output file names,
such as specified by e.g. the -s option, relative to these
directories.
With -multi, the system number is appended to the run input
and each output filename, for instance topol.tpr becomes
topol0.tpr, topol1.tpr etc.
The number of nodes per system is the total number of nodes
divided by the number of systems.
One use of this option is for NMR refinement: when distance
or orientation restraints are present these can be ensemble averaged
over all the systems.

With -replex replica exchange is attempted every given number
of steps. The number of replicas is set with the -multi or
-multidir option, described above.
All run input files should use a different coupling temperature,
the order of the files is not important. The random seed is set with
-reseed. The velocities are scaled and neighbor searching
is performed after every exchange.

Finally some experimental algorithms can be tested when the
appropriate options have been given. Currently under
investigation are: polarizability and X-ray bombardments.

The option -membed does what used to be g\_membed, i.e. embed
a protein into a membrane. The data file should contain the options
that where passed to g\_membed before. The -mn and -mp
both apply to this as well.

The option -pforce is useful when you suspect a simulation
crashes due to too large forces. With this option coordinates and
forces of atoms with a force larger than a certain value will
be printed to stderr.

Checkpoints containing the complete state of the system are written
at regular intervals (option -cpt) to the file -cpo,
unless option -cpt is set to -1.
The previous checkpoint is backed up to state\_prev.cpt to
make sure that a recent state of the system is always available,
even when the simulation is terminated while writing a checkpoint.
With -cpnum all checkpoint files are kept and appended
with the step number.
A simulation can be continued by reading the full state from file
with option -cpi. This option is intelligent in the way that
if no checkpoint file is found, Gromacs just assumes a normal run and
starts from the first step of the .tpr file. By default the output
will be appending to the existing output files. The checkpoint file
contains checksums of all output files, such that you will never
loose data when some output files are modified, corrupt or removed.
There are three scenarios with -cpi:

\* no files with matching names are present: new output files are written

\* all files are present with names and checksums matching those stored
in the checkpoint file: files are appended

\* otherwise no files are modified and a fatal error is generated

With -noappend new output files are opened and the simulation
part number is added to all output file names.
Note that in all cases the checkpoint file itself is not renamed
and will be overwritten, unless its name does not match
the -cpo option.

With checkpointing the output is appended to previously written
output files, unless -noappend is used or none of the previous
output files are present (except for the checkpoint file).
The integrity of the files to be appended is verified using checksums
which are stored in the checkpoint file. This ensures that output can
not be mixed up or corrupted due to file appending. When only some
of the previous output files are present, a fatal error is generated
and no old output files are modified and no new output files are opened.
The result with appending will be the same as from a single run.
The contents will be binary identical, unless you use a different number
of nodes or dynamic load balancing or the FFT library uses optimizations
through timing.

With option -maxh a simulation is terminated and a checkpoint
file is written at the first neighbor search step where the run time
exceeds -maxh\*0.99 hours.

When mdrun receives a TERM signal, it will set nsteps to the current
step plus one. When mdrun receives an INT signal (e.g. when ctrl+C is
pressed), it will stop after the next neighbor search step
(with nstlist=0 at the next step).
In both cases all the usual output will be written to file.
When running with MPI, a signal to one of the mdrun processes
is sufficient, this signal should not be sent to mpirun or
the mdrun process that is the parent of the others.

When mdrun is started with MPI, it does not run niced by default.

### Files

| option | filename | type | description |
| --- | --- | --- | --- |
| **-s** | topol.tpr | Input | Run input file: tpr tpb tpa |
| **-o** | traj.trr | Output | Full precision trajectory: trr trj cpt |
| **-x** | traj.xtc | Output, Opt. | Compressed trajectory (portable xdr format) |
| **-cpi** | state.cpt | Input, Opt. | Checkpoint file |
| **-cpo** | state.cpt | Output, Opt. | Checkpoint file |
| **-c** | confout.gro | Output | Structure file: gro g96 pdb etc. |
| **-e** | ener.edr | Output | Energy file |
| **-g** | md.log | Output | Log file |
| **-dhdl** | dhdl.xvg | Output, Opt. | xvgr/xmgr file |
| **-field** | field.xvg | Output, Opt. | xvgr/xmgr file |
| **-table** | table.xvg | Input, Opt. | xvgr/xmgr file |
| **-tabletf** | tabletf.xvg | Input, Opt. | xvgr/xmgr file |
| **-tablep** | tablep.xvg | Input, Opt. | xvgr/xmgr file |
| **-tableb** | table.xvg | Input, Opt. | xvgr/xmgr file |
| **-rerun** | rerun.xtc | Input, Opt. | Trajectory: xtc trr trj gro g96 pdb cpt |
| **-tpi** | tpi.xvg | Output, Opt. | xvgr/xmgr file |
| **-tpid** | tpidist.xvg | Output, Opt. | xvgr/xmgr file |
| **-ei** | sam.edi | Input, Opt. | ED sampling input |
| **-eo** | edsam.xvg | Output, Opt. | xvgr/xmgr file |
| **-j** | wham.gct | Input, Opt. | General coupling stuff |
| **-jo** | bam.gct | Output, Opt. | General coupling stuff |
| **-ffout** | gct.xvg | Output, Opt. | xvgr/xmgr file |
| **-devout** | deviatie.xvg | Output, Opt. | xvgr/xmgr file |
| **-runav** | runaver.xvg | Output, Opt. | xvgr/xmgr file |
| **-px** | pullx.xvg | Output, Opt. | xvgr/xmgr file |
| **-pf** | pullf.xvg | Output, Opt. | xvgr/xmgr file |
| **-ro** | rotation.xvg | Output, Opt. | xvgr/xmgr file |
| **-ra** | rotangles.log | Output, Opt. | Log file |
| **-rs** | rotslabs.log | Output, Opt. | Log file |
| **-rt** | rottorque.log | Output, Opt. | Log file |
| **-mtx** | nm.mtx | Output, Opt. | Hessian matrix |
| **-dn** | dipole.ndx | Output, Opt. | Index file |
| **-multidir** | rundir | Input, Opt., Mult. | Run directory |
| **-membed** | membed.dat | Input, Opt. | Generic data file |
| **-mp** | membed.top | Input, Opt. | Topology file |
| **-mn** | membed.ndx | Input, Opt. | Index file |

### Other options

| option | type | default | description |
| --- | --- | --- | --- |
| **-[no]h** | bool | no | Print help info and quit || **-[no]version** | bool | no | Print version info and quit || **-nice** | int | 0 | Set the nicelevel || **-deffnm** | string |  | Set the default filename for all file options || **-xvg** | enum | xmgrace | xvg plot formatting: xmgrace, xmgr or none || **-[no]pd** | bool | no | Use particle decompostion || **-dd** | vector | 0 0 0 | Domain decomposition grid, 0 is optimize || **-ddorder** | enum | interleave | DD node order: interleave, pp\_pme or cartesian || **-npme** | int | -1 | Number of separate nodes to be used for PME, -1 is guess || **-nt** | int | 0 | Total number of threads to start (0 is guess) || **-ntmpi** | int | 0 | Number of thread-MPI threads to start (0 is guess) || **-ntomp** | int | 0 | Number of OpenMP threads per MPI process/thread to start (0 is guess) || **-ntomp\_pme** | int | 0 | Number of OpenMP threads per MPI process/thread to start (0 is -ntomp) || **-pin** | enum | auto | Fix threads (or processes) to specific cores: auto, on or off || **-pinoffset** | int | 0 | The starting logical core number for pinning to cores; used to avoid pinning threads from different mdrun instances to the same core || **-pinstride** | int | 0 | Pinning distance in logical cores for threads, use 0 to minimize the number of threads per physical core || **-gpu\_id** | string |  | List of GPU id's to use || **-[no]ddcheck** | bool | yes | Check for all bonded interactions with DD || **-rdd** | real | 0 | The maximum distance for bonded interactions with DD (nm), 0 is determine from initial coordinates || **-rcon** | real | 0 | Maximum distance for P-LINCS (nm), 0 is estimate || **-dlb** | enum | auto | Dynamic load balancing (with DD): auto, no or yes || **-dds** | real | 0.8 | Minimum allowed dlb scaling of the DD cell size || **-gcom** | int | -1 | Global communication frequency || **-nb** | enum | auto | Calculate non-bonded interactions on: auto, cpu, gpu or gpu\_cpu || **-[no]tunepme** | bool | yes | Optimize PME load between PP/PME nodes or GPU/CPU || **-[no]testverlet** | bool | no | Test the Verlet non-bonded scheme || **-[no]v** | bool | no | Be loud and noisy || **-[no]compact** | bool | yes | Write a compact log file || **-[no]seppot** | bool | no | Write separate V and dVdl terms for each interaction type and node to the log file(s) || **-pforce** | real | -1 | Print all forces larger than this (kJ/mol nm) || **-[no]reprod** | bool | no | Try to avoid optimizations that affect binary reproducibility || **-cpt** | real | 15 | Checkpoint interval (minutes) || **-[no]cpnum** | bool | no | Keep and number checkpoint files || **-[no]append** | bool | yes | Append to previous output files when continuing from checkpoint instead of adding the simulation part number to all file names || **-nsteps** | int | -2 | Run this number of steps, overrides .mdp file option || **-maxh** | real | -1 | Terminate after 0.99 times this time (hours) || **-multi** | int | 0 | Do multiple simulations in parallel || **-replex** | int | 0 | Attempt replica exchange periodically with this period (steps) || **-nex** | int | 0 | Number of random exchanges to carry out each exchange interval (N^3 is one suggestion). -nex zero or not specified gives neighbor replica exchange. || **-reseed** | int | -1 | Seed for replica exchange, -1 is generate a seed || **-[no]ionize** | bool | no | Do a simulation including the effect of an X-Ray bombardment on your system |

---

http://www.gromacs.org  
gromacs@gromacs.org
